# Circular RNA hsa_circ_0000467 promotes colorectal cancer progression by promoting eIF4A3-mediated c-Myc translation

**DOI:** 10.1186/s12943-024-02052-5

**Published:** 2024-07-31

**Authors:** Xianjie Jiang, Mingjing Peng, Qiang Liu, Qiu Peng, Linda Oyang, Shizhen Li, Xuemeng Xu, Mengzhou Shen, Jiewen Wang, Haofan Li, Nayiyuan Wu, Shiming Tan, Jinguan Lin, Longzheng Xia, Yanyan Tang, Xia Luo, Qianjin Liao, Yujuan Zhou

**Affiliations:** 1 Hunan Key Laboratory of Cancer Metabolism, Hunan Cancer Hospital and the Affiliated Cancer Hospital of Xiangya School of Medicine, Central South University, 283 Tongzipo Road, Changsha, Hunan 410013 China; 2 Hunan Engineering Research Center of Tumor organoids Technology and application, Public Service Platform of Tumor organoids Technology, 283 Tongzipo Road, Changsha, Hunan 410013 China; 3 University of South China, Hengyang, Hunan 421001 China

**Keywords:** Colorectal cancer, Growth, Metastasis, hsa_circRNA_0000467, eIF4A3, c-Myc, Translation

## Abstract

**Background:**

Colorectal cancer (CRC) is the second most common malignant tumor worldwide, and its incidence rate increases annually. Early diagnosis and treatment are crucial for improving the prognosis of patients with colorectal cancer. Circular RNAs are noncoding RNAs with a closed-loop structure that play a significant role in tumor development. However, the role of circular RNAs in CRC is poorly understood.

**Methods:**

The circular RNA hsa_circ_0000467 was screened in CRC circRNA microarrays using a bioinformatics analysis, and the expression of hsa_circ_0000467 in CRC tissues was determined by in situ hybridization. The associations between the expression level of hsa_circ_0000467 and the clinical characteristics of CRC patients were evaluated. Then, the role of hsa_circ_0000467 in CRC growth and metastasis was assessed by CCK8 assay, EdU assay, plate colony formation assay, wound healing assay, and Transwell assay in vitro and in a mouse model of CRC in vivo. Proteomic analysis and western blotting were performed to investigate the effect of hsa_circ_0000467 on c-Myc signaling. Polysome profiling, RT‒qPCR and dual-luciferase reporter assays were performed to determine the effect of hsa_circ_0000467 on c-Myc translation. RNA pull-down, RNA immunoprecipitation (RIP) and immunofluorescence staining were performed to assess the effect of hsa_circ_0000467 on eIF4A3 distribution.

**Results:**

In this study, we found that the circular RNA hsa_circ_0000467 is highly expressed in colorectal cancer and is significantly correlated with poor prognosis in CRC patients. In vitro and in vivo experiments revealed that hsa_circ_0000467 promotes the growth and metastasis of colorectal cancer cells. Mechanistically, hsa_circ_0000467 binds eIF4A3 to suppress its nuclear translocation. In addition, it can also act as a scaffold molecule that binds eIF4A3 and c-Myc mRNA to form complexes in the cytoplasm, thereby promoting the translation of c-Myc. In turn, c-Myc upregulates its downstream targets, including the cell cycle-related factors cyclin D2 and CDK4 and the tight junction-related factor ZEB1, and downregulates E-cadherin, which ultimately promotes the growth and metastasis of CRC.

**Conclusions:**

Our findings revealed that hsa_circRNA_0000467 plays a role in the progression of CRC by promoting eIF4A3-mediated c-Myc translation. This study provides a theoretical basis and molecular target for the diagnosis and treatment of CRC.

**Supplementary Information:**

The online version contains supplementary material available at 10.1186/s12943-024-02052-5.

## Introduction

Colorectal cancer (CRC) is a frequently occurring malignant tumor of the lower gastrointestinal tract that is usually found in the lining of the colon and rectum [1]. The International Agency for Research on Cancer estimated that CRC accounted for approximately 1,931,000 newly diagnosed tumors (approximately 10% of all tumors) worldwide in 2020, making it the second most commonly diagnosed tumor worldwide. Additionally, as approximately 935,000 CRC-related deaths occurred, CRC ranked third in the number of cancer-related deaths in 2020. It is expected that the number of new cases of CRC will increase to 2.5 million worldwide by 2035 [2, 3]. The effectiveness of CRC treatment has greatly improved due to continuous improvements in surgical instruments and techniques and the gradual standardization of preoperative neoadjuvant therapy and postoperative comprehensive therapy. As a result, the 5-year survival rate of early-stage CRC patients exceeds 90%. However, the 5-year survival rate for all patients with CRC is only approximately 60%. Additionally, for those who have already developed metastasis at the time of diagnosis, the 5-year survival rate is less than 20% [4, 5]. CRC tumorigenesis is a complex process influenced by genetic, environmental, dietary, and lifestyle factors and alterations in gene expression [3, 6].

Circular RNAs (circRNAs) are a type of noncoding RNA that have a closed loop structure. These RNAs lack a cap at the 5’ end and a poly(A) tail at the 3’ end, which means they are less susceptible to RNA exonucleases and are more stable than linear RNAs. Cells contain high levels of circRNAs [7], and recent studies have shown that circRNAs play a crucial role in tumorigenesis and development. They regulate various biological processes, including tumor cell growth, invasion, migration, angiogenesis, and immune escape [8–11]. In CRC, circRNA_0000392 can act as a miRNA sponge to adsorb miR-193a-5p, upregulate PIK3R3 expression and promote malignant progression [12]. Exosomal circLPAR1 can enter CRC cells via endocytosis and bind to the eIF3 protein, thereby blocking the interaction between METTL3 and eIF3h. This process suppresses the translation of BRD4 and ultimately inhibits CRC progression [13]. The ciRS-122/miR-122/PKM2 signaling axis promotes glycolysis in CRC, which promotes the resistance of cancer cells to the chemotherapeutic agent oxaliplatin [14]. Furthermore, circRNAs play a significant role in angiogenesis in CRC. For instance, circ3823 can stimulate angiogenesis in CRC by establishing the circ3823/miR-30c-5p/TCF7 signaling axis through a ceRNA mechanism [15]. However, our current understanding of the role of cyclic RNA in CRC is not comprehensive, and therefore, additional studies are needed.

In this study, we screened hsa_circ_0000467 (abbreviated as circ467), a circRNA that is highly expressed in CRC, using CRC circRNA microarrays from the Gene Expression Omnibus (GEO) database. The high expression of circ467 in CRC tissues was confirmed by in situ hybridization. Furthermore, high circ467 expression was significantly associated with poor prognosis in CRC patients. Using in vitro and in vivo experiments, we found that circ467 binds to eIF4A3 and suppresses its nuclear translocation; moreover, circ467 can form a complex with eIF4A3 and c-Myc mRNA in the cytoplasm, which promotes c-Myc translation and activates c-Myc signaling. This leads to the upregulation of the cell cycle-associated factors cyclin D2 and CDK4, as well as the cellular tight junction-associated factor ZEB1. Additionally, this process downregulates E-cadherin expression, which ultimately promotes the growth and metastasis of CRC. This study reveals the role and mechanism of hsa_circRNA_0000467 in the malignant progression of CRC, deepens our understanding of the roles of circRNAs in the malignant progression of CRC, and provides a new theoretical basis and molecular target for the diagnosis and treatment of CRC.

## Results

1 Circ467 expression is significantly upregulated in CRC, and high circ467 expression is inversely correlated with the prognosis in CRC patients.

**Fig. 1:**
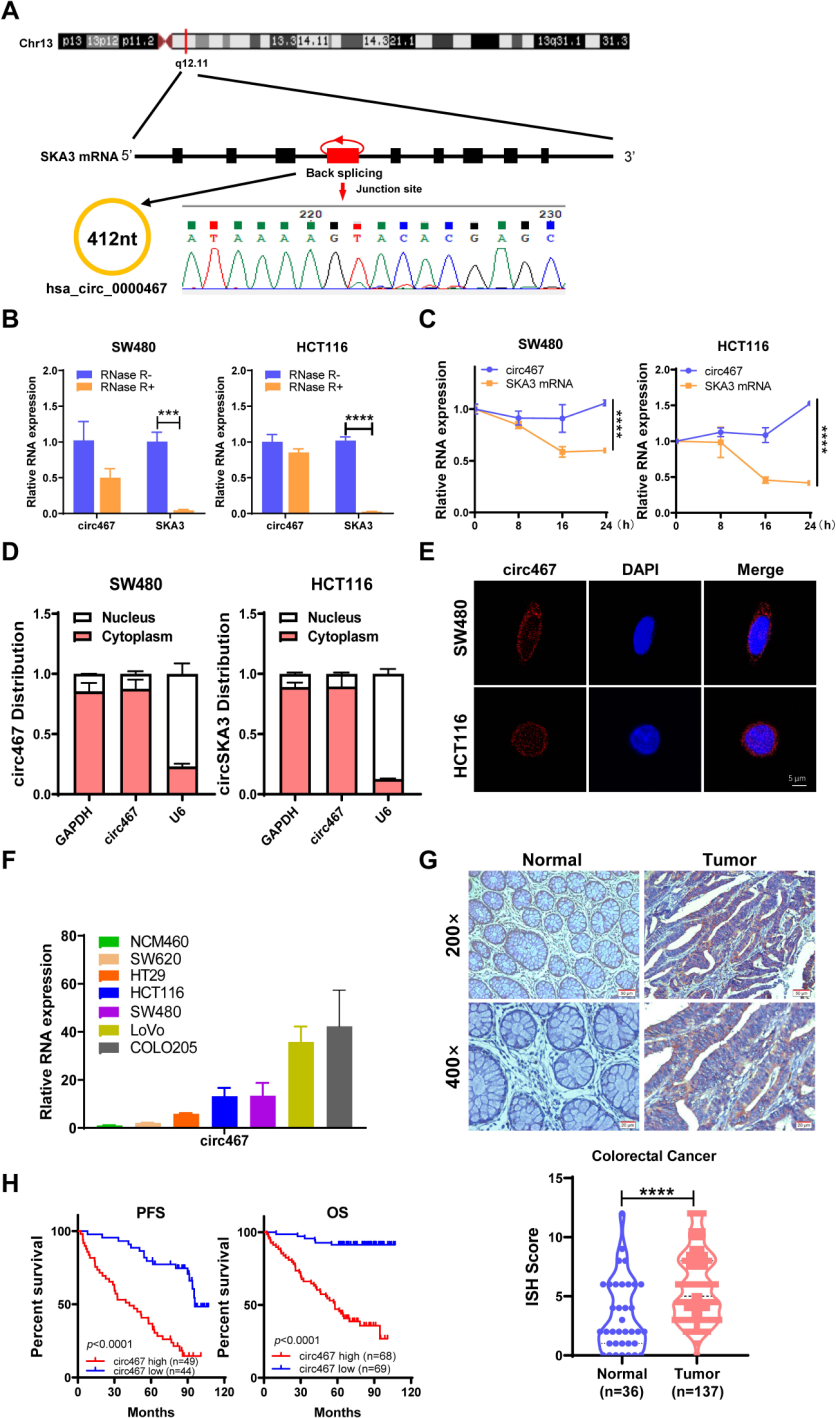
Circ467 is highly expressed in CRC and is inversely correlated with the prognosis in CRC patients. (**A**) Schematic diagram of circ467 generation. (**B**) SW480 and HCT116 cells were treated with RNase R, and the stability of circ467 was assessed using RT‒qPCR. (**C**) SW480 and HCT116 cells were treated with actinomycin D for the indicated times, and the stability of circ467 was assessed using RT‒qPCR. (**D**) The cellular localization of circ467 was assessed by RNA nucleoplasmic separation assays. (**E**) The intracellular distribution of circ467 was assessed by fluorescence in situ hybridization (scale bar = 5 μm). (**F**) circ467 expression in CRC cells and NCM460 cells (immortalized colorectal epithelial cells) was assessed using RT‒qPCR. (**G**) Representative results showing circ467 expression in 137 CRC tissues and 36 adjacent nontumor tissues according to in situ hybridization (upper panel); circ467 was highly expressed in CRC tissues (lower panel). Magnification: 200×, scale bar = 50 μm; magnification: 400×; scale bar = 20 μm. (**H**) Kaplan‒Meier analysis of overall survival and progression-free survival of CRC patients grouped according to circ467 expression. The data shown are representative images or are expressed as the mean ± SD of each group from three separate experiments or a single experiment (for the in vivo studies) (***, *p* < 0.001; ****, *p* < 0.0001 vs. control; Student’s t test)

To explore the key circRNAs involved in CRC development, we screened for significantly differentially expressed circRNAs in CRC by analyzing the CRC circRNA expression microarrays GSE138589 and GSE142837 from the GEO database. We found a total of 186 differentially expressed circRNAs (fold change ≥ 2) in the GSE138589 cohort and a total of 43 differentially expressed circRNAs (fold change ≥ 2) in the GSE142837 cohort (Figure S1A). After observing the overlapping differentially expressed circRNAs in both microarrays, four circRNAs were found to be differentially expressed in both datasets (Figure S1B). The expression of these 4 circRNAs in CRC cells was subsequently examined, and it was found that hsa_circRNA_0000467 had the highest expression in CRC cells (Figure S1C). Sanger sequencing and circBase database analysis revealed that hsa_circRNA_0000467 is generated via reverse splicing of exon 4 of SKA3; henceforth, this circRNA is abbreviated as circ467 (Fig. 1A). Circ467 is a circRNA that is stabilized in CRC cells after treatment with RNase R and actinomycin D (Fig. 1B and C). RNA nucleoplasmic separation and RNA fluorescence in situ hybridization assays confirmed that circ467 was located mainly in the cytoplasm of CRC cells (Fig. 1D and E). In addition, we assessed the expression of circ467 in CRC cell lines and found that circ467 was expressed at low levels in the immortalized colorectal epithelial cell line NCM460 and was significantly upregulated in CRC cell lines, which is consistent with the results of the microarray analyses. We found that circ467 was most highly expressed in the LoVo and COLO205 cell lines, was expressed at relatively low levels in the SW620 and HT29 cell lines, and was moderately expressed in the SW480 and HCT116 cell lines (Fig. 1F). We next assessed the expression of circ467 in 137 CRC tissue samples and 36 cancer-adjacent normal colorectal epithelial tissue samples using in situ hybridization and found that circ467 was significantly upregulated in CRC tissues compared with normal cancer-adjacent colorectal epithelial tissues (Fig. 1G). The prognosis of CRC patients with low circ467 expression was relatively good, while the prognosis of CRC patients with high circ467 expression was poor, which indicates that circ467 expression is significantly negatively correlated with the prognosis of CRC patients (Fig. 1H). In addition, we analyzed the correlation between circ467 expression and the clinical features of patients and found that circ467 expression was not correlated with age, sex or extent of tumor histology grading (*p* > 0.05) (Figures S2A, S2B and S2C); however, circ467 expression was significantly correlated with TNM stage and the occurrence of metastasis in patients with CRC (*p* < 0.05) (Figures S2D and S2E). The results above revealed that circ467 is a highly expressed circRNA in CRC and that circ467 expression was significantly negatively correlated with the prognosis of CRC patients, which suggests that circ467 may play an important role in the malignant progression of CRC.

### Circ467 promotes CRC growth in vitro and in vivo

**Fig. 2:**
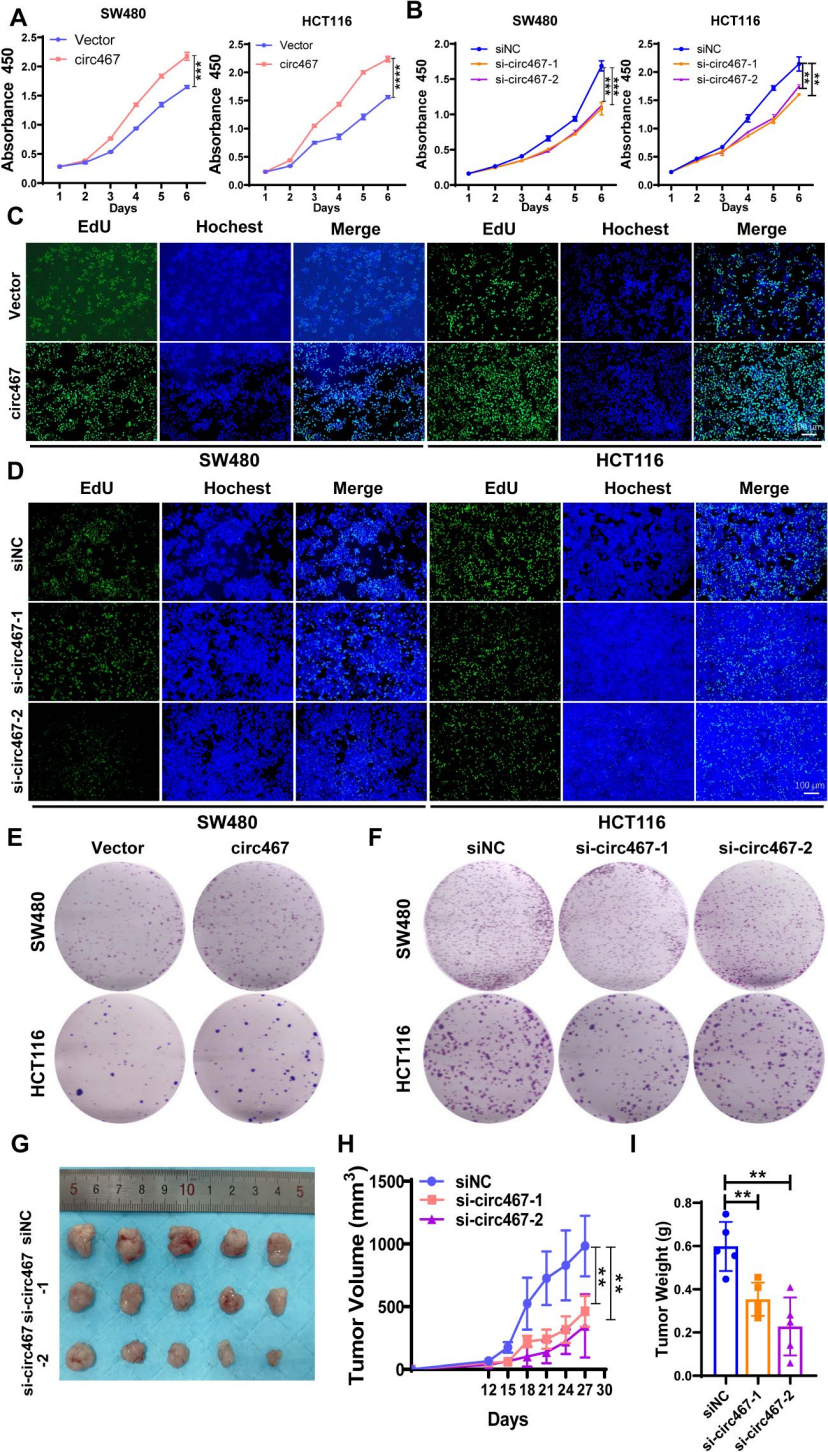
Circ467 promotes the growth of CRC cells in vitro and in vivo **A**, **B**. The proliferation of SW480 and HCT116 cells after circ467 overexpression or knockdown was assessed by CCK-8 assay. **C**, **D**. The proliferation of SW480 and HCT116 cells after circ467 overexpression or knockdown was assessed by EdU assay. **E**, **F**. Colony formation assays were performed in SW480 and HCT116 cells after circ467 overexpression or knockdown. **G**. Images of tumors formed in nude mice after transplantation of HCT116 cells transfected with circ467 siRNAs. **H**. Statistical analysis of the growth curves of tumors formed in nude mice after transplantation of HCT116 cells transfected with circ467 siRNAs. I. Statistical analysis of the weight of xenograft tissues in nude mice after transplantation of HCT116 cells transfected with circ467 siRNAs. The data shown are representative images or are expressed as the mean ± SD of each group from three experiments or a single experiment (for the in vivo studies) (**, *p* < 0.01; ***, *p* < 0.001; ****, *p* < 0.0001 vs. control; Student’s t test)

The growth and metastasis of tumor cells are important factors in the malignant progression of CRC. To assess the role of circ467 in CRC growth, we overexpressed or knocked down circ467 in SW480 and HCT116 cells (Figures S3A and S3B), after which a CCK-8 assay was performed to evaluate the growth of these cells. The results showed that circ467 overexpression promoted the growth of CRC cells, while circ467 knockdown inhibited the growth of CRC cells (Fig. 2A and B). Furthermore, we investigated the role of circ467 in CRC cell proliferation using EdU staining. Our findings showed that circ467 overexpression promoted CRC cell proliferation, whereas circ467 knockdown inhibited CRC cell proliferation (Fig. 2C and D and S3C). Additionally, we investigated the role of circ467 in CRC cell colony formation using a plate colony formation assay. The results showed that circ467 overexpression increased the clonogenicity of CRC cells, while circ467 knockdown reduced clonogenicity (Fig. 2E and F and S3D). These results confirmed that circ467 significantly promotes the growth of CRC cells in vitro.

To assess the role of circ467 on CRC growth in vivo, we subcutaneously injected circ467-knockdown CRC cells into mice to establish a mouse model. Subsequent experiments showed that the growth of subcutaneous xenograft tumors in nude mice was significantly inhibited by the knockdown of circ467. The tumor volume in the circ467 knockdown group was significantly smaller than that in the control group (Fig. 2G and I). In addition, using in situ hybridization and immunohistochemical analysis of subcutaneous tumor tissues, we discovered that the proportion of proliferating cells in CRC tissues in the circ467 knockdown group was significantly lower than that in the control group (Figure S3E). These results confirmed that circ467 can also significantly promote CRC growth in vivo.

### Circ467 promotes the metastasis of CRC in vitro and in vivo

**Fig. 3:**
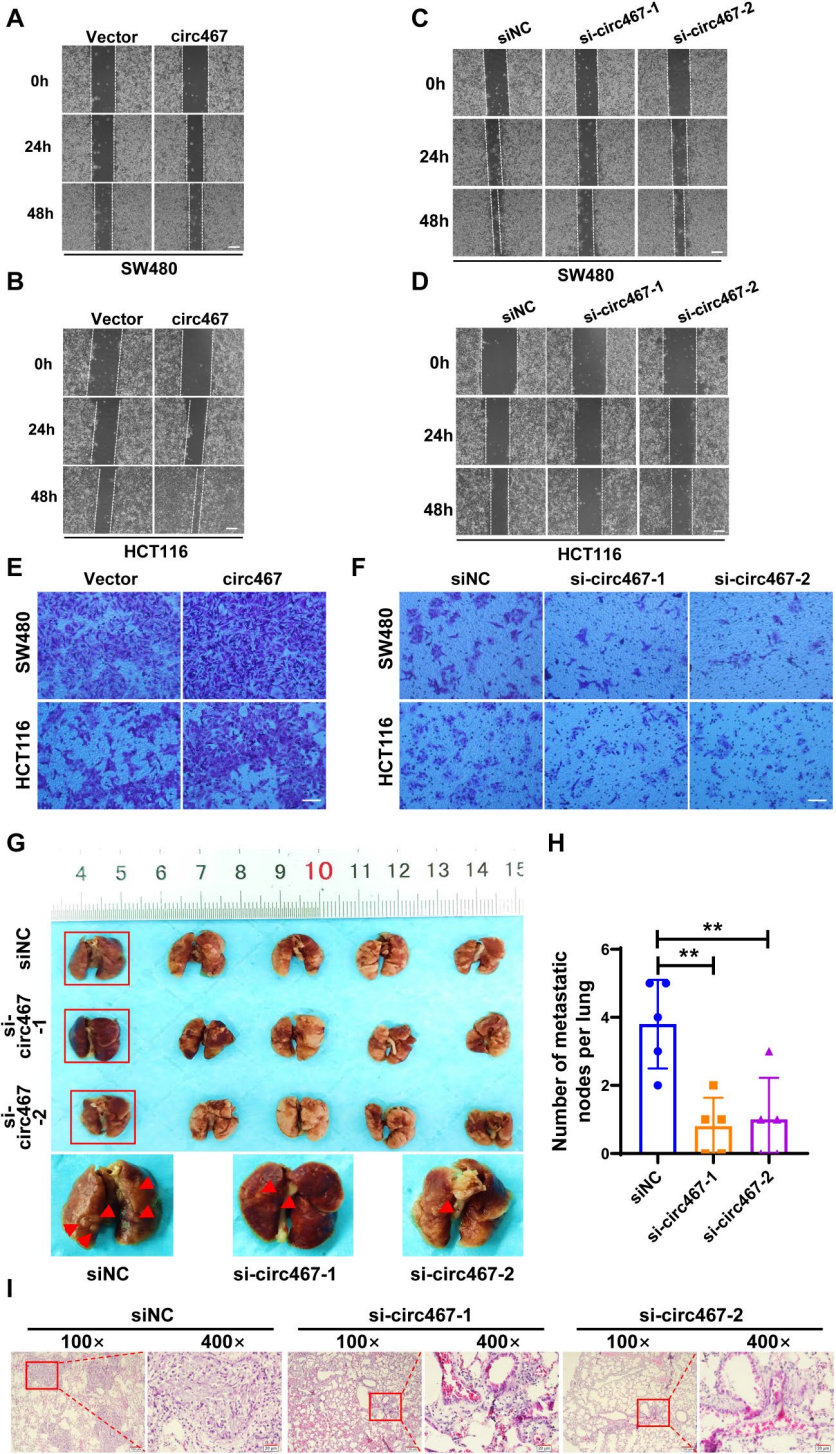
Circ467 promotes the metastasis of CRC in vitro and in vivo **A**, **B**. The migration of SW480 and HCT116 cells after circ467 overexpression was assessed using a wound healing assay. Scale bar = 200 μm. **C**, **D**. The migration of SW480 and HCT116 cells after circ467 knockdown was assessed using a wound healing assay. Scale bar = 200 μm. **E**, **F**. The invasiveness of SW480 and HCT116 cells after circ467 overexpression or knockdown was assessed by Transwell assay. Scale bar = 50 μm. **G**. Images of visible nodules on the lung surface after HCT116 cells in which circ467 was knocked down were injected into the tail vein of nude mice (*n* = 5); arrows show visible nodules on the lung surface. **H**. Quantification of metastatic nodules on the lung surface. **I**. Representative images of H&E-stained metastatic lung nodules after circ467 knockdown. Magnification = 100×, scale bar = 100 μm; magnification = 400×; scale bar = 20 μm. The data shown are representative images or are expressed as the mean ± SD of each group from a single experiment (for the in vivo studies) (**, *p* < 0.01 vs. control; Student’s t test)

To investigate the role of circ467 in CRC metastasis, we overexpressed or knocked down circ467 in CRC cells. Wound healing and Transwell assays were performed to evaluate the migration and invasion of CRC cells, respectively. The wound healing assay showed that circ467 overexpression facilitated the migration of CRC cells, whereas circ467 knockdown significantly inhibited the migration of CRC cells (Fig. 3A and D, S4A and S4B). Similar results were obtained with the Transwell assay (Fig. 3E and F and S4C). We also investigated the role of circ467 in the invasion and metastasis of CRC cells in vivo by generating a tail vein-to-lung metastasis model in nude mice. The results showed that circ467 knockdown reduced the metastasis of CRC cells to the lungs. The number of metastatic nodules in the lung tissues of mice in the circ467 knockdown group was significantly lower than that in the control group (Fig. 3G and H). H&E staining of the mouse lung tissues confirmed the presence of significantly fewer lung tumors in the circ467 knockdown group than in the control group, and the tumors were significantly smaller (Fig. 3I). These data confirmed that circ467 can promote CRC metastasis in vivo.

### Circ467 may regulate the cell cycle and tight junction signaling by activating c-Myc signaling

**Fig. 4:**
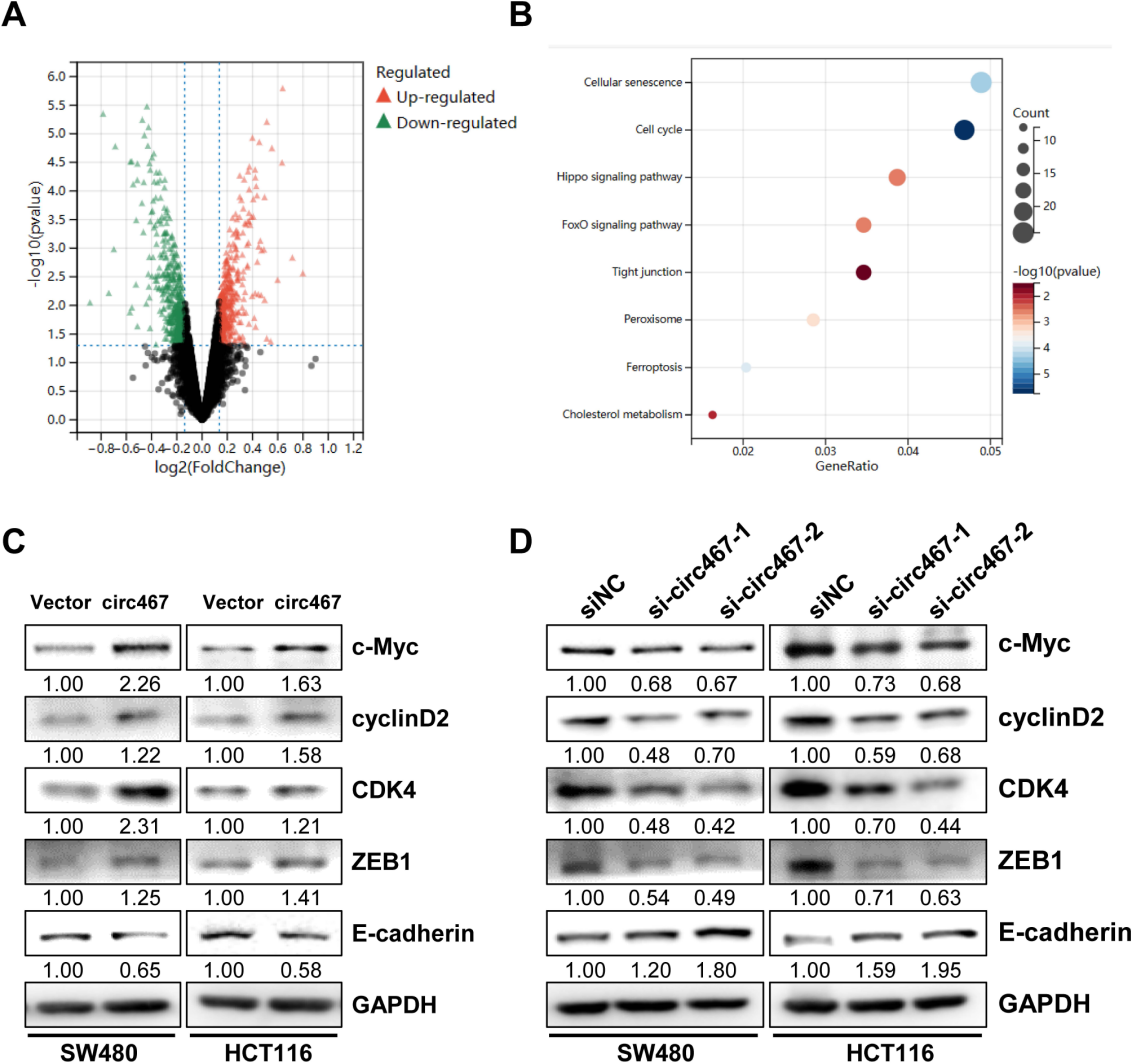
Circ467 can activate c-Myc signaling (**A**) Volcano plot showing differentially expressed proteins in control and circ467-overexpressing HCT116 cells. (**B**) The principal biological processes regulated by circ467 were determined through KEGG enrichment analysis of the differentially expressed proteins between control and circ467-overexpressing HCT116 cells. (**C**) The expression of c-Myc, cyclin D2, CDK4, ZEB1, and E-cadherin in SW480 and HCT116 cells after circ467 overexpression was assessed by western blotting; GAPDH served as an internal control. (**D**) The expression of c-Myc, cyclin D2, CDK4, ZEB1, and E-cadherin in SW480 and HCT116 cells after circ467 knockdown was assessed by western blotting; GAPDH served as an internal control

To investigate the mechanism of action of circ467 in CRC growth and metastasis, a proteomic analysis was performed to examine the protein expression profile of circ467-overexpressing HCT116 cells (Fig. 4A). KEGG analysis of the differentially expressed proteins revealed that overexpression of circ467 resulted in abnormal cell cycle progression and cellular tight junction signaling in CRC cells (Fig. 4B). Several studies have demonstrated a significant correlation between the cell cycle and cell growth. Additionally, aberrant tight junction signaling has been shown to lead directly to tumor cell invasion and metastasis [16, 17]. C-Myc is a crucial protein in the cell cycle and in tight junction signaling. Studies have demonstrated that c-Myc can regulate cell growth by modulating cell cycle-related target molecules, including cyclin D2, CDK4, and cyclin E [18, 19]. Furthermore, c-Myc can regulate cell‒cell junctions by modulating the expression of molecules related to epithelial–mesenchymal transition (EMT), such as E-cadherin and ZEB1. This modulation can have an impact on the migration and invasion of tumor cells [20–22]. The proteomics analysis suggested that circ467 overexpression significantly upregulated c-Myc protein expression. Therefore, circ467 may regulate the cell cycle and cellular tight junction status of CRC cells by activating the c-Myc signaling pathway. To assess the role of circ467 in c-Myc signaling, we overexpressed and knocked down circ467 in SW480 and HCT116 cells. These experiments showed that circ467 overexpression increased the protein expression of c-Myc, cyclin D2 and CDK4, which are cell cycle-related proteins. Additionally, c-Myc upregulated the expression of the tight junction-related molecule ZEB1 and downregulated the expression of E-cadherin. Conversely, circ467 knockdown induced the opposite effects (Fig. 4C and D). These results confirmed that circ467 may regulate the cell cycle and cellular tight junction signaling in CRC by activating c-Myc signaling.

### Circ467 promotes growth, migration and invasion by promoting c-Myc signaling

**Fig. 5:**
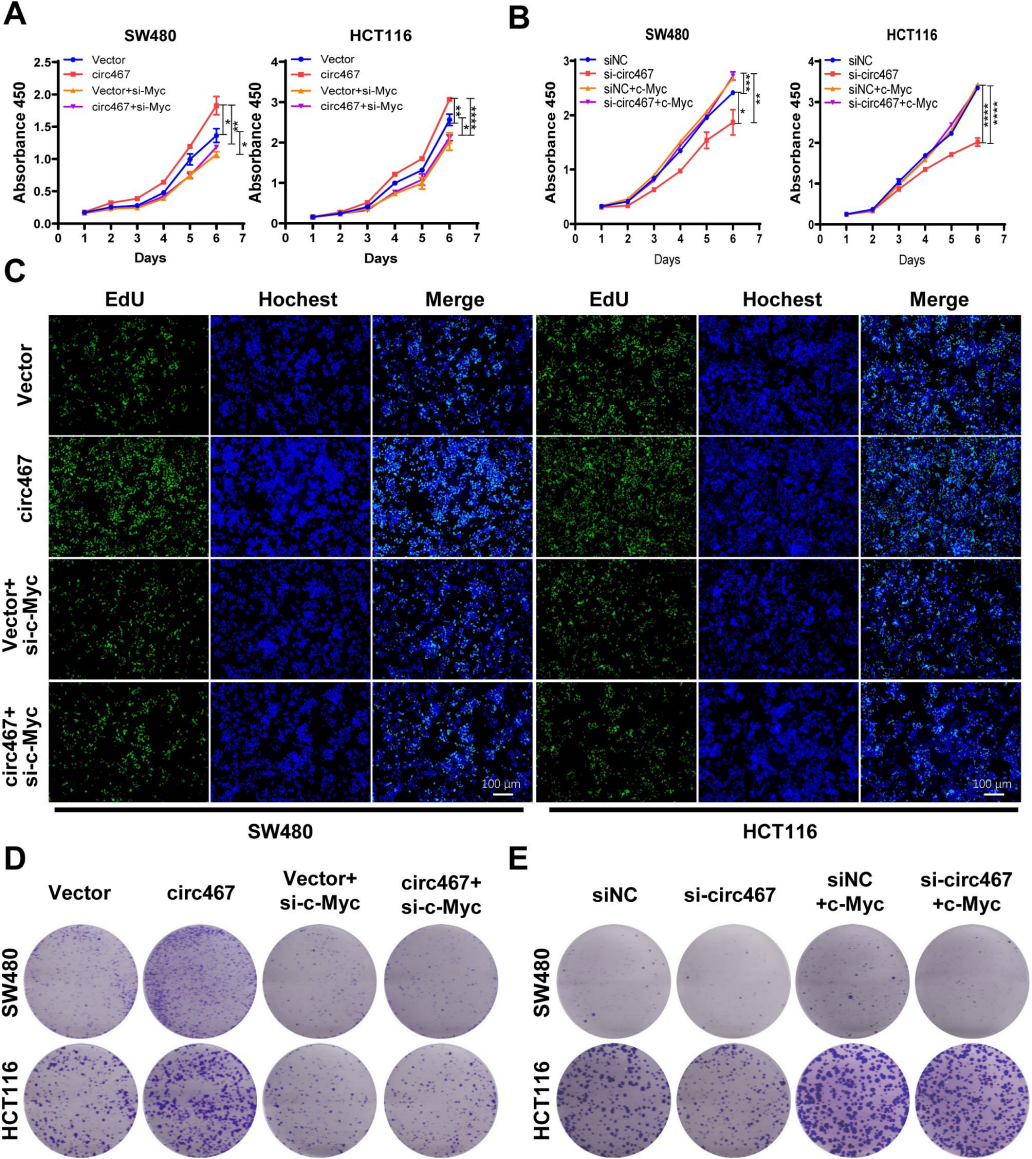
Circ467 promotes the growth of CRC cells by upregulating c-Myc. (**A**) CCK-8 assays were performed to assess the growth of SW480 and HCT116 cells after cotransfection of circ467 plasmids and c-Myc siRNAs. (**B**) CCK-8 assays were performed to assess the growth of SW480 and HCT116 cells after cotransfection of circ467 siRNAs and c-Myc plasmids. (**C**) EdU assays were performed to assess the growth of SW480 and HCT116 cells after cotransfection of circ467 plasmids and c-Myc siRNAs. (**D**) Transwell assays were performed to assess the growth of SW480 and HCT116 cells after cotransfection of circ467 plasmids and c-Myc siRNAs. (**E**) Transwell assays were performed to assess the growth of SW480 and HCT116 cells after cotransfection of circ467 siRNAs and c-Myc plasmids. The data shown are representative images or are expressed as the mean ± SD of each group from three separate experiments (*, *p* < 0.05; **, *p* < 0.01; ***, *p* < 0.001; ****, *p* < 0.0001 vs. control; Student’s t test)

**Fig. 6:**
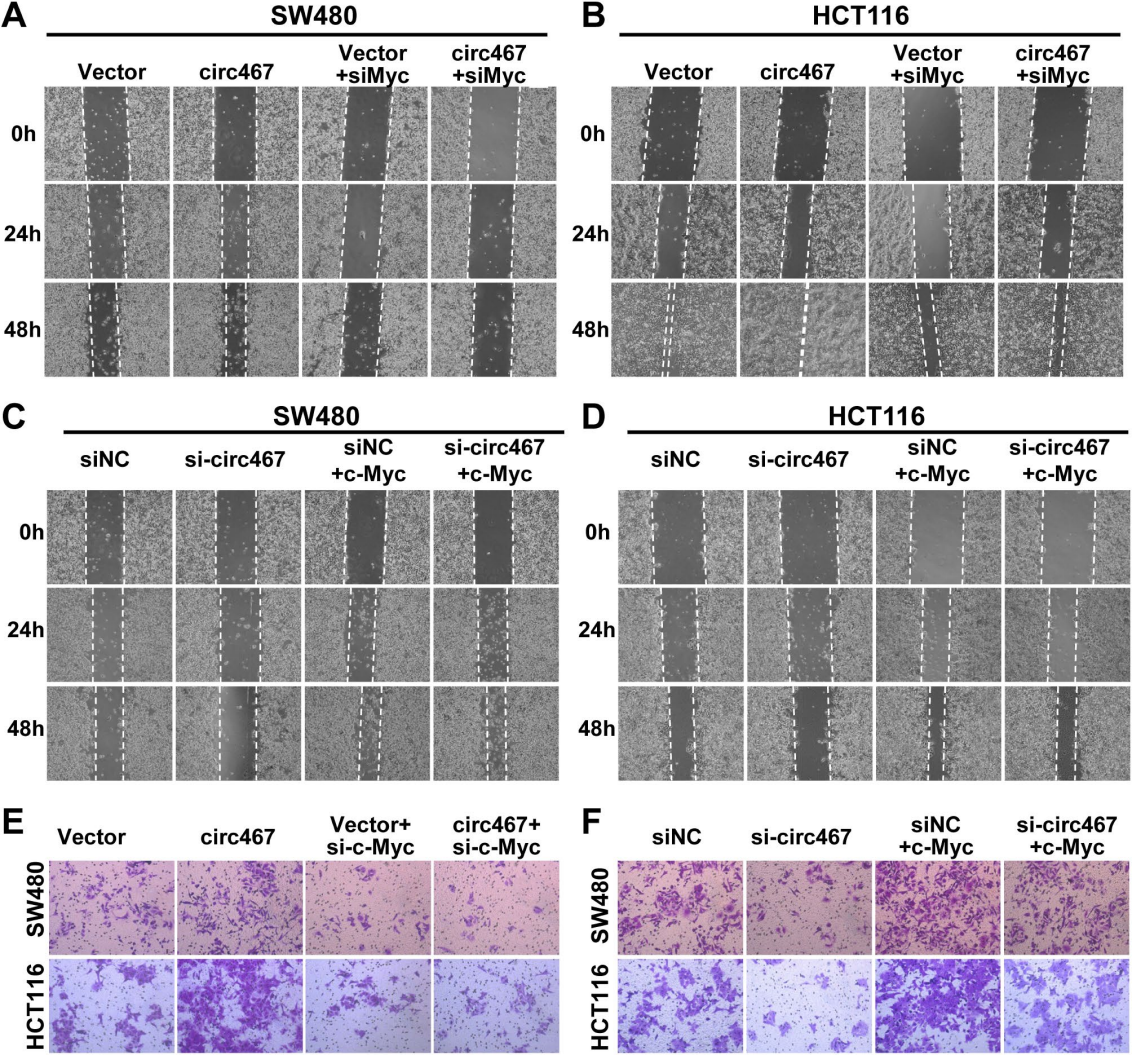
Circ467 promotes the migration and invasion of CRC cells by upregulating c-Myc. **A**, **B**. Wound healing assays were performed using SW480 and HCT116 cells after cotransfection with circ467 plasmids and c-Myc siRNAs. **C**, **D**. Wound healing assays were performed using SW480 and HCT116 cells after cotransfection with circ467 siRNAs and c-Myc plasmids. **E**. Transwell assays were performed using SW480 and HCT116 cells after cotransfection with circ467 plasmids and c-Myc siRNAs. **F**. Transwell assays were performed using SW480 and HCT116 cells after cotransfection with circ467 siRNAs and c-Myc plasmids. The data shown are representative images of three separate experiments from each group

To investigate whether circ467 promotes CRC cell growth, migration and invasion via c-Myc, we overexpressed circ467 in CRC cells and knocked down c-Myc or overexpressed c-Myc with concomitant knockdown of circ467 (Figures S5A and S5B) and determined the growth of CRC cells using a CCK-8 assay, EdU assay and plate colony formation assay. CCK-8 assays showed that simultaneous circ467 overexpression and c-Myc silencing attenuated the circ467 overexpression-induced increase in CRC cell growth. Conversely, c-Myc overexpression attenuated the inhibitory effect of circ467 silencing on CRC cell growth (Fig. 5A and B). Consistent results were also obtained in the EdU staining assay (Fig. 5C, S5C and S5D). Plate colony formation assays confirmed that c-Myc knockdown in CRC cells overexpressing circ467 significantly attenuated the circ467 overexpression-induced increase in colony formation (Fig. 5D and E and S5E). These findings indicated that circ467 promotes the proliferation of CRC cells primarily by increasing the expression of c-Myc. A wound healing assay revealed that silencing c-Myc in cells overexpressing circ467 significantly attenuated the ability of circ467 to promote CRC cell migration. Conversely, silencing circ467 expression along with the restoration of c-Myc expression induced the opposite effects (Fig. 6A and D, S6A and S6B). Similar results were observed in a Transwell assay (Fig. 6E and F and S6C). The results above demonstrate that circ467 may promote the growth, migration and invasion of CRC cells by activating the c-Myc signaling pathway.

### Circ467 promotes c-Myc translation

**Fig. 7:**
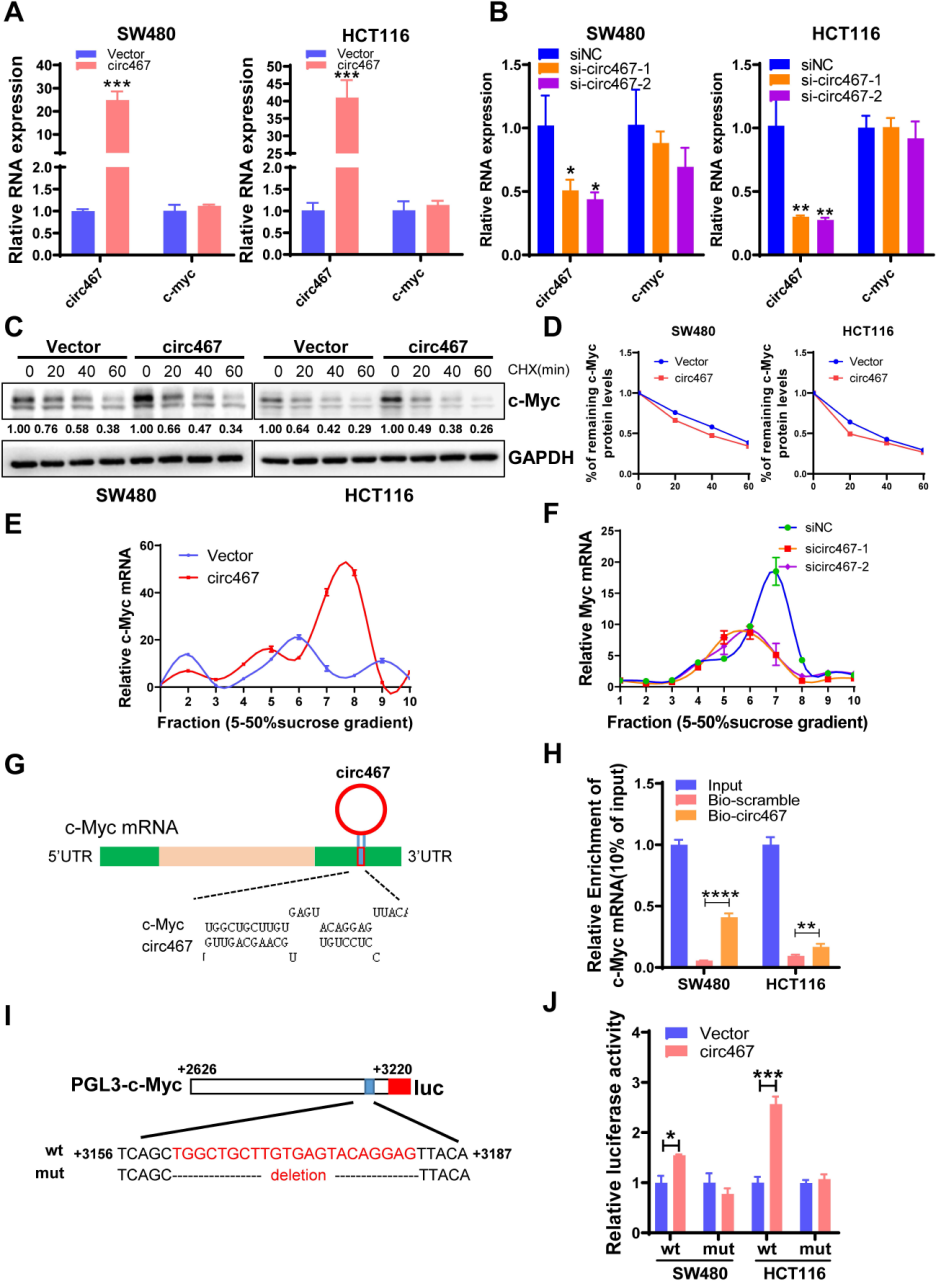
Circ467 may increase the translation efficiency of c-Myc. **A** The expression of circ467 and c-Myc in SW480 and HCT116 cells after circ467 overexpression was assessed using RT‒qPCR. **B** The expression of circ467 and c-Myc in SW480 and HCT116 cells after circ467 knockdown was assessed using RT‒qPCR. **C** The expression of c-Myc in SW480 and HCT116 cells after circ467 overexpression and cycloheximide (CHX) treatment was assessed by western blotting. **D** Quantification of c-Myc protein degradation according to C. **E**, **F**. Polysomes in cytoplasmic extracts from HCT116 cells after circ467 overexpression or knockdown were fractionated by sucrose gradients, and the relative c-Myc mRNA expression level in the gradient fractions was analyzed using RT‒qPCR. **G** Diagram showing the interaction between circ467 and c-Myc mRNA. **H** The interaction between circ467 and c-Myc mRNA in SW480 and HCT116 cells was evaluated by RNA pull-down using an anti-circ467 probe, followed by RT‒qPCR analysis of c-Myc expression. **I** Schematic diagram of the construction of the wild-type and mutant c-Myc 3’ UTR reporters. **J** c-Myc reporter activity in SW480 and HCT116 cells cotransfected with the circ467 plasmid and potential c-Myc reporters (PGL3-c-Myc-wt or PGL3-c-Myc-mut) was assessed using a dual luciferase reporter assay. The data shown are representative images or are expressed as the mean ± SD of each group from three separate experiments (*, *p* < 0.05; **, *p* < 0.01; ***, *p* < 0.001; ****, *p* < 0.0001 vs. control, Student’s t test)

To investigate the molecular mechanism by which circ467 promotes c-Myc expression, we overexpressed or knocked down circ467 in SW480 and HCT116 cells. The effect of circ467 on the level of c-Myc mRNA was detected by RT‒qPCR. The results showed that circ467 did not affect the expression of c-Myc mRNA (Fig. 7A and B). These data suggest that circ467 does not regulate c-Myc at the transcriptional level. Next, we added cyclohexamide (CHX) to SW480 and HCT116 cells overexpressing circ467 and examined the effect of circ467 on the rate of c-Myc protein degradation using western blotting. The results indicated that circ467 does not significantly impact the degradation rate of the c-Myc protein (Fig. 7C and D). Current evidence indicates that circular RNAs regulate protein expression by affecting the transcription, protein degradation, or translation efficiency of mRNAs. The results of our study indicate that circ467 does not affect c-Myc transcription or protein stability. Therefore, these findings suggested that circ467 regulates c-Myc at the translational level. To investigate the effect of circ467 on c-Myc translation, we performed a ribosome isolation assay coupled with RT‒qPCR in HCT116 CRC cells in which circ467 was either overexpressed or knocked down. Overexpression of circ467 promoted the translation of c-Myc mRNA, while knockdown of circ467 suppressed the translation of c-Myc mRNA (Fig. 7E and F). These results revealed that circ467 can promote the translation of c-Myc. Bioinformatics prediction analysis revealed that circ467 can bind to the 3’-UTR of c-Myc mRNA, and RNA pull-down experiments showed that circ467 can interact with c-Myc mRNA in SW480 and HCT116 cells (Fig. 7G and H). To investigate whether circ467 promotes the translation of c-Myc by binding to the 3’ UTR of c-Myc mRNA, we constructed wild-type and mutant c-Myc 3’-UTR dual-luciferase reporter gene vectors in which the binding site for circ467 was deleted, we then performed a dual-luciferase reporter gene assay to assess the effects on reporter activity. Circ467 overexpression increased the activity of the wild-type reporter gene expression vectors but had no significant effect on the activity of the mutant reporter gene expression vectors (Fig. 7I and J). The experimental results suggested that circ467 may increase the protein expression of c-Myc by promoting the translation of c-Myc mRNA.

### Circ467 upregulates c-Myc by binding to eIF4A3

**Fig. 8:**
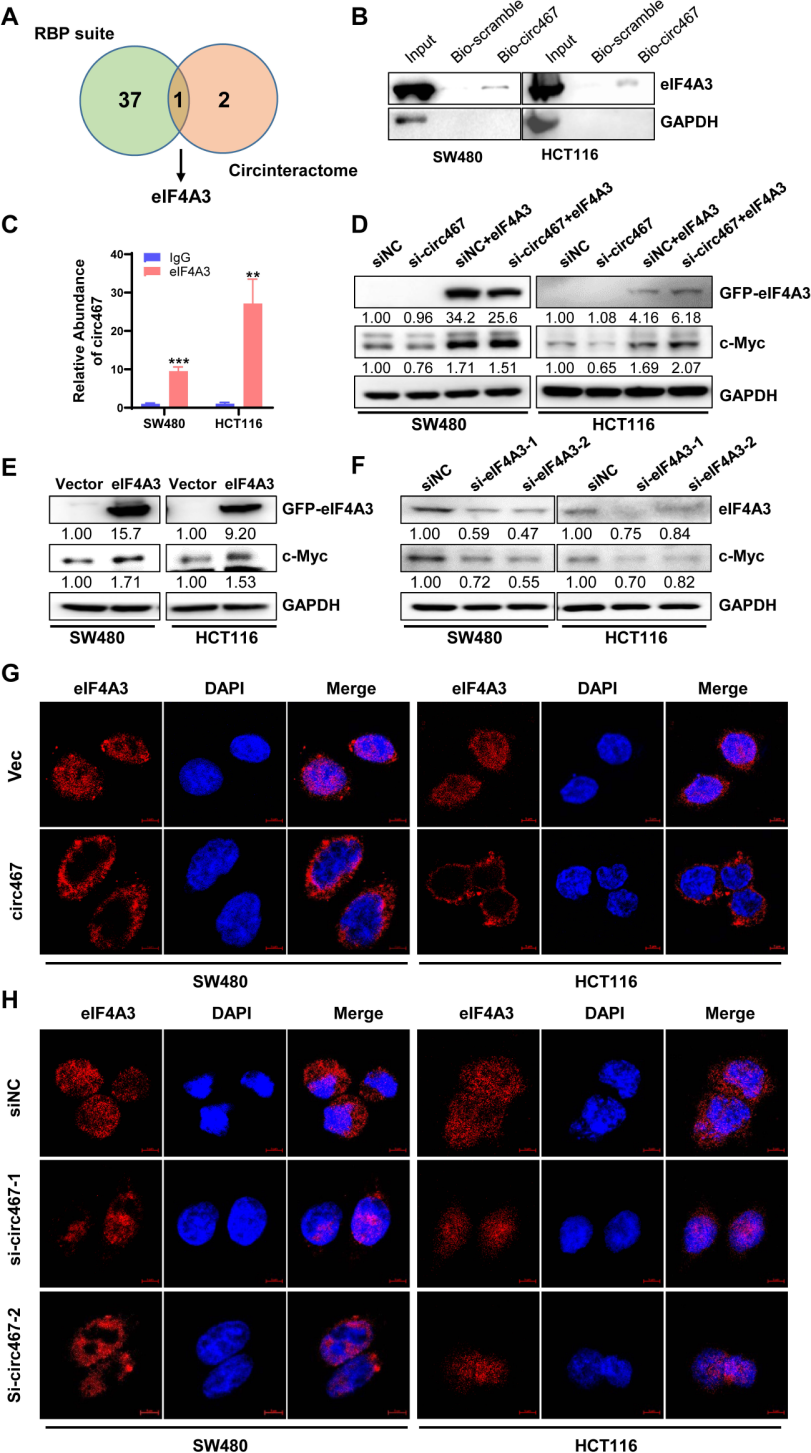
Circ467 upregulates c-Myc by binding to eIF4A3 and inhibiting its nuclear translocation **A** Venn diagram indicating that eIF4A3 is an RNA-binding protein of circ467 according to both RNA-binding protein databases. **B** The interaction between circ467 and eIF4A3 in SW480 and HCT116 cells was evaluated by RNA pull-down using an anti-circ467 probe, followed by western blotting analysis of eIF4A3. **C** The interaction between eIF4A3 and circ467 in SW480 and HCT116 cells was evaluated by RNA immunoprecipitation using an anti-eIF4A3 antibody, followed by RT‒qPCR. **D** The expression of GFP-eIF4A3 and c-Myc in SW480 and HCT116 cells after cotransfection of circ467 siRNAs and GFP-eIF4A3 plasmids was evaluated by western blotting. **E**, **F** The expression of eIF4A3 and c-Myc in SW480 and HCT116 cells after GFP-eIF4A3 overexpression or eIF4A3 knockdown was assessed by western blotting. **G**, **H**. The distribution of eIF4A3 in SW480 and HCT116 cells after circ467 overexpression or knockdown was assessed by immunofluorescence staining. The data shown are representative images or are expressed as the mean ± SD of each group from three separate experiments (**, *p* < 0.01; ***, *p* < 0.001 vs. control; Student’s t test)

Existing studies do not indicate that circular RNAs can directly regulate protein translation. Therefore, we hypothesized that circ467 may regulate the translation of c-Myc by binding to other proteins. Analysis of the RNA-binding protein database RBPsuite and the circInteractome database revealed that eIF4A3 may interact with circ467 (Fig. 8A). EIF4A3 is a member of the translation initiation family and regulates the translation of related genes [23, 24]. Circ467 may act as a scaffolding molecule by binding to eIF4A3 and c-Myc mRNA, thereby facilitating the translation of c-Myc mRNA. RNA pull-down experiments demonstrated that circ467 binds to the eIF4A3 protein (Fig. 8B), and RIP experiments confirmed that eIF4A3 also binds to circ467 (Fig. 8C). eIF4A3 overexpression in CRC cells reversed the downregulation of c-Myc induced by circ467 knockdown (Fig. 8D). Upregulation of c-Myc expression was observed in CRC cells overexpressing eIF4A3, whereas knockdown of eIF4A3 resulted in downregulation of c-Myc protein expression (Fig. 8E and F). These results indicated that circ467 activates c-Myc signaling by binding to eIF4A3, thereby promoting the translation of c-Myc, which in turn upregulates c-Myc protein expression. Several studies have demonstrated that eIF4A3, a member of the translation initiation factor family, regulates protein translation in the cytoplasm. Additionally, eIF4A3 functions as a splicing factor in the nucleus, where it participates in the alternative splicing of numerous genes [25, 26]. To assess the role of circ467 in the regulation of eIF4A3 function, we overexpressed and knocked down circ467 in CRC cells. Subsequently, we assessed the distribution of eIF4A3 in these cells using cellular immunofluorescence analysis. Circ467 overexpression led to the retention of eIF4A3 in the cytoplasm, whereas circ467 knockdown resulted in the aggregation of high levels of eIF4A3 protein in the nucleus (Fig. 8G and H). Overall, the results indicated that circ467 binds to eIF4A3, sequestering it in the cytoplasm, and acts as a scaffolding molecule to form a complex by binding to eIF4A3 and c-Myc mRNA, which in turn facilitates c-Myc translation.

## Discussion

**Fig. 9:**
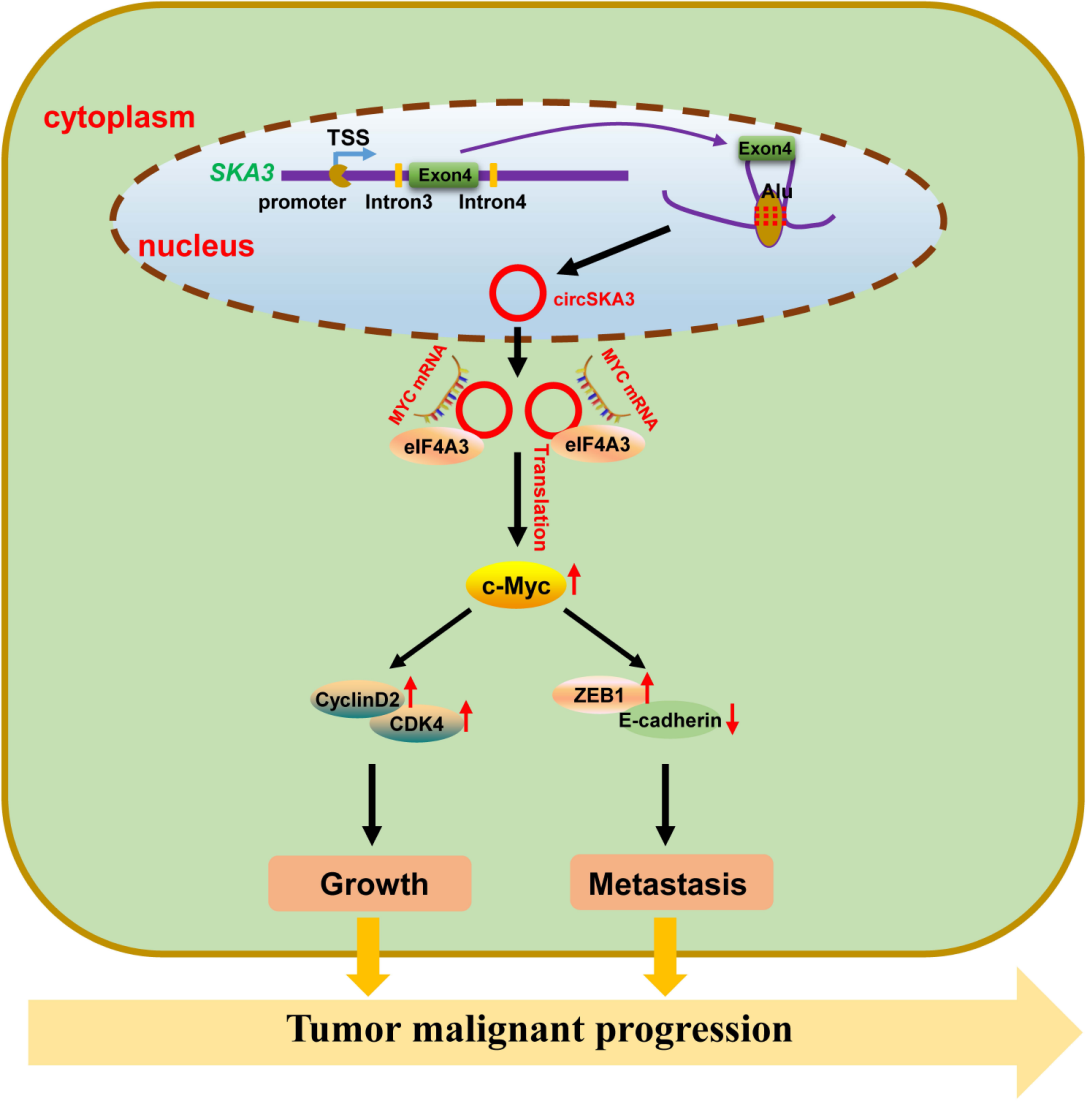
Schematic representation of the possible mechanism of circ467 in the malignant progression of CRC

CircRNAs are a type of noncoding RNA and have been extensively researched in recent years. CircRNAs can be found in large quantities in cells, saliva, serum and urine and are characterized by a closed loop structure, which makes them much more stable than linear RNAs [27–29]. Numerous studies have reported that circRNAs play important roles in tumor development [30]. In this study, we found that circ467 is highly expressed in CRC tissues and that high circ467 expression is significantly associated with poor prognosis in CRC patients. Circ467 is a circRNA derived from exon 4 of the SKA3 gene located on chromosome 13 (13q12.11). This circRNA is generated through reverse splicing under the influence of alternative splicing factors and is composed of 412 nucleotides. Research has shown that in gastric cancer, circ467 can act as an RNA sponge that binds miR-326-3p to promote the malignant progression of gastric cancer [31]. Additionally, studies have shown that circ467 expression is associated with the prognosis of gastric cancer patients, which indicates its potential as a prognostic biomarker [32]. It has also been reported that circ467 promotes the malignant progression of CRC cells, but its specific molecular mechanism is still unclear [33]. This study revealed that circ467 can bind eIF4A3 and suppress its nuclear translocation. Furthermore, circ467 can serve as a scaffold to form a complex with eIF4A3 and c-Myc mRNA, which promotes the translation of c-Myc and the expression of the cell cycle-associated proteins cyclin D2 and CDK4, located downstream of c-Myc. In this process, the expression of the cellular tight junction-associated protein ZEB1 is upregulated, while the expression of E-cadherin is downregulated, ultimately promoting the growth, invasion and migration of CRC cells (Fig. 9). This finding provides new insight into the mechanism by which circ467 regulates CRC development.

The transcription factor c-Myc is crucial for the transcription of approximately 10–15% of all human genes. This protein plays a significant role in various cellular processes, such as cell proliferation, tumor metastasis, angiogenesis, immune escape, glucose metabolism, and lipid metabolism [34–36]. Rapid tumor cell growth, invasion, and metastasis are important factors in the malignant progression of tumors [37]. The cell cycle refers to the complete process from the beginning of cell division to the completion of the next division, and an abnormality in the cell cycle directly affects the rate of cell growth [16]. The cell cycle in tumors is modulated by c-Myc, which regulates the expression of downstream target molecules, including cyclin D1/2, cyclin E1, CDK4, cdc25A, and E2F1, and promotes tumor growth [38, 39]. Proteomic analysis in this study revealed abnormal cell cycle signaling in CRC cells overexpressing circ467, and further investigation revealed that circ467 promoted the growth of CRC cells by upregulating the protein expression of c-Myc, which subsequently induced the downstream expression of cell cycle-associated cyclin D2 and CDK4; these results indicate the molecular mechanism by which circ467 promotes the growth of CRC cells. Tumor metastasis is closely related to the strength of intercellular tight junctions, as the loosening of intercellular junctions in tumors increases the invasion and migration potential of tumor cells [40]. E-cadherin is a crucial cellular adhesion molecule, and as its expression increases, tighter cell connections are formed, while downregulation of E-cadherin expression facilitates tumor cell metastasis [41, 42]. ZEB1 is a member of the zinc-finger E-box binding protein family and serves as a marker for epithelial–mesenchymal transition (EMT). ZEB1 can promote EMT by inhibiting the expression of E-cadherin or promoting the expression of other molecules [43, 44]. Proteomic analysis indicated that circ467 affects tight junction signaling, and further studies showed that circ467 upregulates ZEB1 and downregulates E-cadherin by increasing c-Myc protein expression. These findings indicate the molecular mechanism by which circ467 may promote the metastasis of CRC. Furthermore, proteomic analysis revealed that circ467 may be linked to cellular senescence, ferroptosis, and cholesterol metabolism, but these associations require further investigation.

This study revealed that circ467 promotes the translation of c-Myc. However, this study did not provide evidence of the direct involvement of circRNAs in translation regulation. Analysis of the RBPsuite and circInteractome databases revealed that circ467 might interact with the translation initiation factor eIF4A3. EIF4A3 is an ATP-dependent RNA deconjugating enzyme and a core component of the exon junction complex (EJC) [45, 46]. The formation of the EJC by eIF4A3 initiates nonsense-mediated mRNA decay, which impacts mRNA levels and regulates protein expression at the translational and posttranslational levels [47]. In addition, eIF4A3 can be used as a marker to evaluate mRNA quality before translation initiation and facilitates the accurate binding of proteins in the translation initiation complex to the intended target mRNAs [48]. Our study revealed that circ467 can bind to and sequester eIF4A3 in the cytoplasm and form a complex with c-Myc mRNA to promote c-Myc protein translation.

Abnormalities in alternative splicing factors are significantly associated with high expression of circRNAs in tumors. eIF4A3, which is a core member of the exon junction complex, regulates the alternative splicing of numerous circRNAs [49, 50]. In gliomas, eIF4A3 induces the expression of circASAP1, which promotes malignant progression and resistance to temozolomide [51]. In cervical cancer, the expression of circ-0087429, which is induced by eIF4A3, reverses the EMT process and inhibits the malignant progression of the disease [25]. In the present study, neither the overexpression nor the knockdown of eIF4A3 significantly affected the expression of circ467 (data not shown). Aberrant expression of variable splicing factors and m6A modification of circRNA molecules are important factors that affect circRNA expression, these mechanisms are also key contributors to the heightened presence of circular RNAs observed in tumor [52, 53]. Further in-depth investigations are needed to determine why circ467 is highly expressed in CRC.

## Conclusion

In summary, our study revealed that circ467 can increase the protein translation efficiency of c-Myc by binding to eIF4A3, which in turn promotes the malignant progression of CRC. This finding is significant because it highlights a previously unknown mechanism of cancer progression. Our work provides new insights into the mechanism of CRC progression and potential therapeutic targets for CRC treatment.

## Materials and methods

### Cell lines

The CRC cell lines SW480 and HCT116 were maintained in our laboratory. The cells were cultured in RIMI-1640 medium supplemented with 10% fetal bovine serum (OriCell, GuangZhou, China), penicillin (100 U/mL), and streptomycin (100 µg/mL) in a humidified incubator under 5% CO2 at 37 °C.

### CRC tissue samples

A total of 137 surgical CRC tissues and 36 adjacent non-tumor tissues were collected from the Affiliated Cancer Hospital of Xiangya School of Medicine, Central South University between 2007 and 2011.Their demographic and clinical characteristics are shown in table S1. These tissues were fixed and paraffin-embedded. These patients were followed up with a mean follow-up of 67.9 mouths (2.29-106.97 mouths). The study was approved by the Joint Ethics Committee of the Central South University. Health Authority and informed consent was obtained from all participants. The diagnosis of all specimens was confirmed by histopathological examination.

### Plasmids, siRNAs and, reagents

pcDNA3.1-circ467, pcDNA3.1-c-Myc and pcDNA3.1-IF4A3-GFP plasmids were purchased from Genechem (Shanghai, China). circ467, c-Myc and EIF4A3 siRNAs were purchased from RiBo Bio (Guangzhou, China). Hiperfect used for siRNA transfection was purchased from Qiagen (USA). TransIT-X2 used for plasmids transfection was purchased from Dakewei (Shenzhen, China).

### RNA isolation and RT-qPCR

Total RNA was isolated using TRIzol reagent (Invitrogen, USA) according to the manufacturer’s instructions. First-strand cDNA was prepared using a reverse transcription kit (Thermofisher, USA) according to the manufacturer’s protocol. SYBR was used for RT-qPCR analysis on the LightCycler 480 (Roche, USA). After reactions were completed, the relative gene expression levels were calculated using the 2^–ΔΔCt^ method, and GAPDH was used as the endogenous control. The primer sequences used are listed in Table S1.

### Western blotting

Total proteins were lysed using RIPA buffer containing a protease inhibitor cocktail (Beyotime Biotechnology, Jiangsu, China). The proteins were separated by 10% SDS-PAGE and transferred onto PVDF membranes (Millipore, Billerica, MA, USA). The membrane was blocked with 5% non-fat milk with TBST for 1 h at room temperature and incubated with the appropriate primary antibodies overnight at 4 °C. After washing with TBST for three times, the membrane was incubated with HRP-labeled secondary antibodies (Abclonal, Wuhan, China) for 2 h at room temperature. The proteins were then examined using ECL reagent (Millipore). The primary antibodies were listed in Table S2.

### Luciferase reporter assay

All luciferase reporter constructs were purchased from Shanghai Genechem, The luciferase activity was examined by the Dual-Luciferase Reporter Assay System (Promega). The relative luciferase signal was presented as firefly luciferase activity normalized to the Renilla luciferase activity.

### RNase R and actinomycin D treatment

RNase R (Geneseed, Guangzhou) treatment experiments were performed according to the manufacturer’s instructions. 2 µg/ml actinomycin D (Sigma, USA) was added to cells at 0 h, 8 h, 16 h and 24 h, Cells were then collected and RNA was extracted for RT-qPCR assay. The primer sequences were listed in table S1.

### Fluorescence in situ hybridization (FISH) and in situ hybridization (ISH)

The FISH experiment was performed according to the manufacturer’s protocol. Briefly, cells were fixed and permeabilized with 0.3% Triton X-100, and then hybridized with anti-circ467 probe overnight at 37℃, and the nuclei were stained with DAPI (Beyotime, Shanghai). For in situ hybridization, formalin-fixed paraffin-embedded CRC tissues and mouse tissues were deparaffinized and rehydrated, RNA fragments were exposed by treatment with 3% H_2_O_2_ for 10 min followed by treatment with pepsin for 20 min, then the tissues were hybridized with anti-circ467 probe overnight at 37℃. The staining was scored according to the staining intensity and the distribution of stained cells as described previous [52].

### Immunofluorescence

Cells seeded in crawls were fixed with 4% paraformaldehyde for 15 min, followed by blocking with 5% BSA for 1 h, and then incubated with appropriate primary antibodies at 4 °C overnight, after three washes with 0.5 M PBS, cells were incubated with appropriate fluorescent secondary antibodies for 1 h at 37 °C. The nucleus was stained with DAPI for 10 min and the cells were photographed under a confocal microscope (Carl Zeiss AG, Germany).

### Wound healing and transwell assay

For the wound healing assay, cells seeded in a 6-well plate were scratched with a 10 µl tip when the cells grew to confluence. Images were then taken under a microscope at 0 h, 24 h, 48 h. For the transwell assay, 60 µL of diluted Matrigel (Corning, USA) was added to a transwell chamber prior to the addition of cells; after the matrix gel had solidified, 600 µL of medium containing 20% FBS was added to the lower chamber and 200 µL of medium (containing 105 cells) without FBS was added to the upper chamber. The chambers were fixed in 4% paraformaldehyde and stained with crystal violet. Three randomly selected areas were photographed under the microscope and the number of cells was counted using Image J software.

### Proteomics analysis

HCT116 cells overexpressing circ467 were subjected to tandem mass tags (TMT) proteomic analysis (Luming Biotechnology, China). Samples were extracted, lysed and labelled with TMT and then prepared for detection by liquid chromatography-mass spectrometry (LC-MS). The differentially expressed proteins were then further used for enrichment analysis.

### Polysome profiling

HCT116 cells after circ467 overexpression or knockdown were treated with 100 µg/mL cycloheximide (CHX, Selleck) for 15 min, then the cells were harvested and lysed with hypotonic lysis buffer (5 mM Tris-HCl, 2.5 mM MgCl_2_, 1.5 mM KCl, 2 mM DTT, 0.5% Triton X-100, 0.5% sodium deoxycholate). Samples were centrifuged at 16,000 g for 10 min at 4℃. For fractionation, cytoplasmic lysates were loaded onto 5-50% sucrose gradients and separated by ultracentrifugation using a SW42 rotor (Beckman) at 32,000 rpm for 3 h at 4℃. Linear sucrose gradients were collected and RNA was extracted from each fraction for RT-qPCR.

### RNA immunoprecipitation (RIP)

RIP was performed by using a Magna RIP kit (Milipore, USA) according to the manufacturer’s protocol. Briefly, cells were harvested, lysed with RIP lysis buffer and incubated with magnetic beads and specific antibodies or IgG antibodies on a rotator overnight at 4 ℃. Magnetic bead immunoprecipitated RNA was purified and enriched for subsequent RT-qPCR analysis.

#### RNA pull down assay

For the RNA pull-down assay, cells were lysed and sonicated, and then the cell lysates were mixed with the biotin-labelled circ467 probe overnight at 4 ℃, Streptavidin affinity magnetic beads were then added to the lysates for 2 h at room temperature. The supernatant washed from the magnetic beads was analysed by Western blotting.

### Animal experiments

Subcutaneous xenograft model: The 4-week-old nu/nu-BALB/c athymic nude mice were purchased from Silaikejingda (Changsha, China). A total of 15 mice were randomly divided into 3 groups (*n* = 5 per group), 5 × 10^6^ HCT116 cells transfected with control or circ467 siRNAs were injected subcutaneously into the respective mice. Tumor growth was observed every three days, and the tumor volume was calculated as was assessed by measuring the largest perpendicular diameters, and the tumor volume was calculated as follows: V = 1/2 × (length) × (width) × (width). After 33 days of subcutaneous inoculation, the mice were euthanized by cervical dislocation and the tumor tissue was excised. The formed tumor masses were removed and weighed. All animal protocols were approved by the Institutional Laboratory of Animal Care and Use Committee of Central South University.

For tail vein injection experiments, a total of 15 mice were randomly divided into 3 groups (*n* = 5 per group). Each mouse was injected via the tail vein with 10^6^ HCT116 cells transfected with circ467 siRNAs. Mice were sacrificed 8 weeks after injection and lung tissues were collected for pathological examination. All animal protocols were approved by the Institutional Animal Care and Use Committee of the Central South University.

### Statistical analysis

Statistical analyses were performed using GraphPad Prism 8.0 software. Differences between groups were analyzed using the Student’s t-test when there were only two groups, or using one-way ANOVA when there were more than two groups. A two-tailed value of *p* < 0.05 was considered statistically significant (*, *p* < 0.05; **, *p* < 0.01; ***, *p* < 0.001; ****, *p* < 0.0001).

### Electronic supplementary material

Below is the link to the electronic supplementary material.

Supplementary Material 1

Supplementary Material 2

Supplementary Material 3

Supplementary Material 4

Supplementary Material 5

Supplementary Material 6

Supplementary Material 7

Supplementary Material 8

Supplementary Material 9

Supplementary Material 10

## Data Availability

No datasets were generated or analysed during the current study.
