# Supplementary figures and images for: Circular RNA hsa_circ_0000467 promotes colorectal cancer progression by promoting eIF4A3-mediated c-Myc translation
Source: Mol Cancer. 2024 Jul 31;23:151. doi: 10.1186/s12943-024-02052-5 (PMC11290134; doi:10.1186/s12943-024-02052-5)

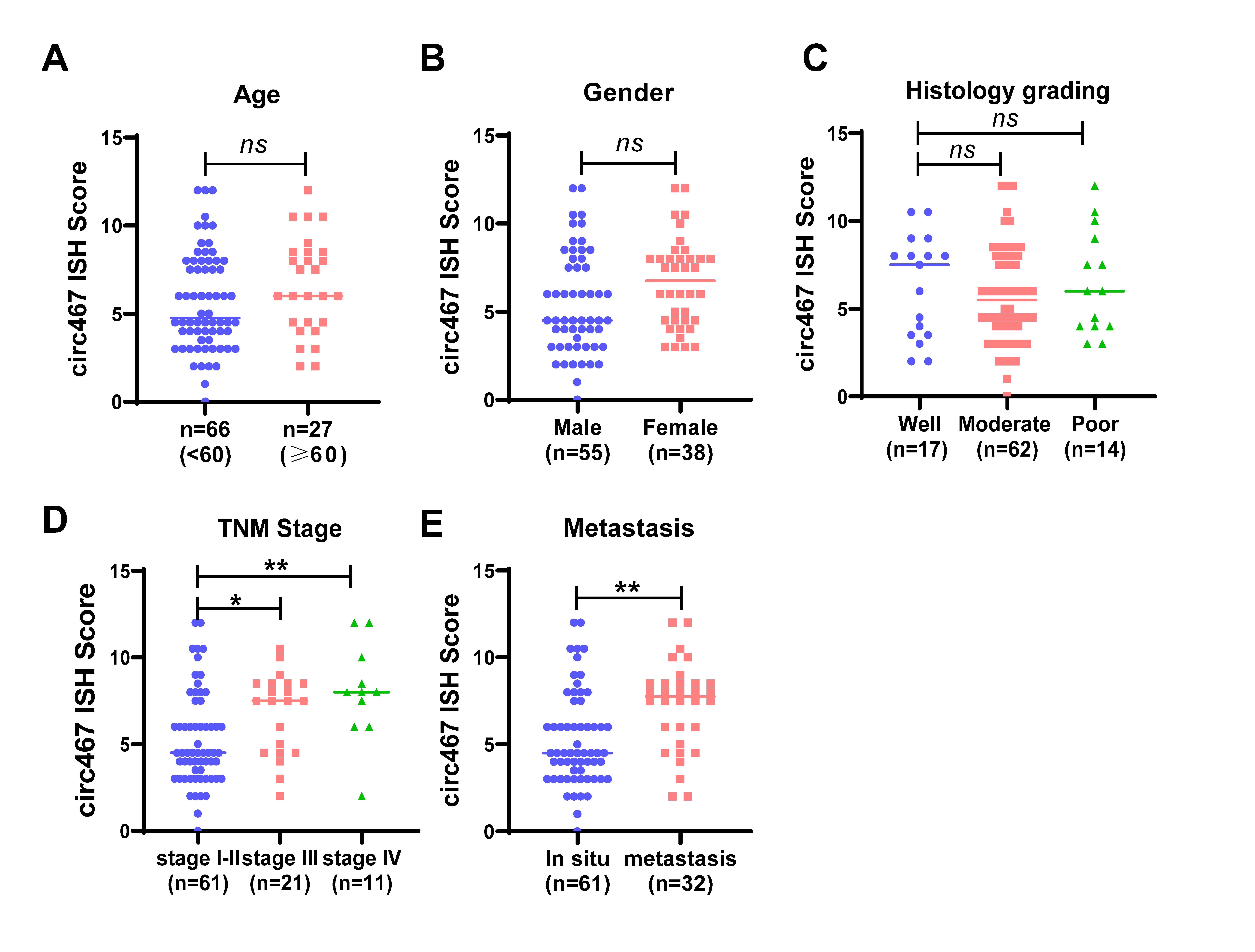

Supplement: Supplementary file 2 — Supplementary Material 2 [file 12943_2024_2052_MOESM2_ESM.jpg]

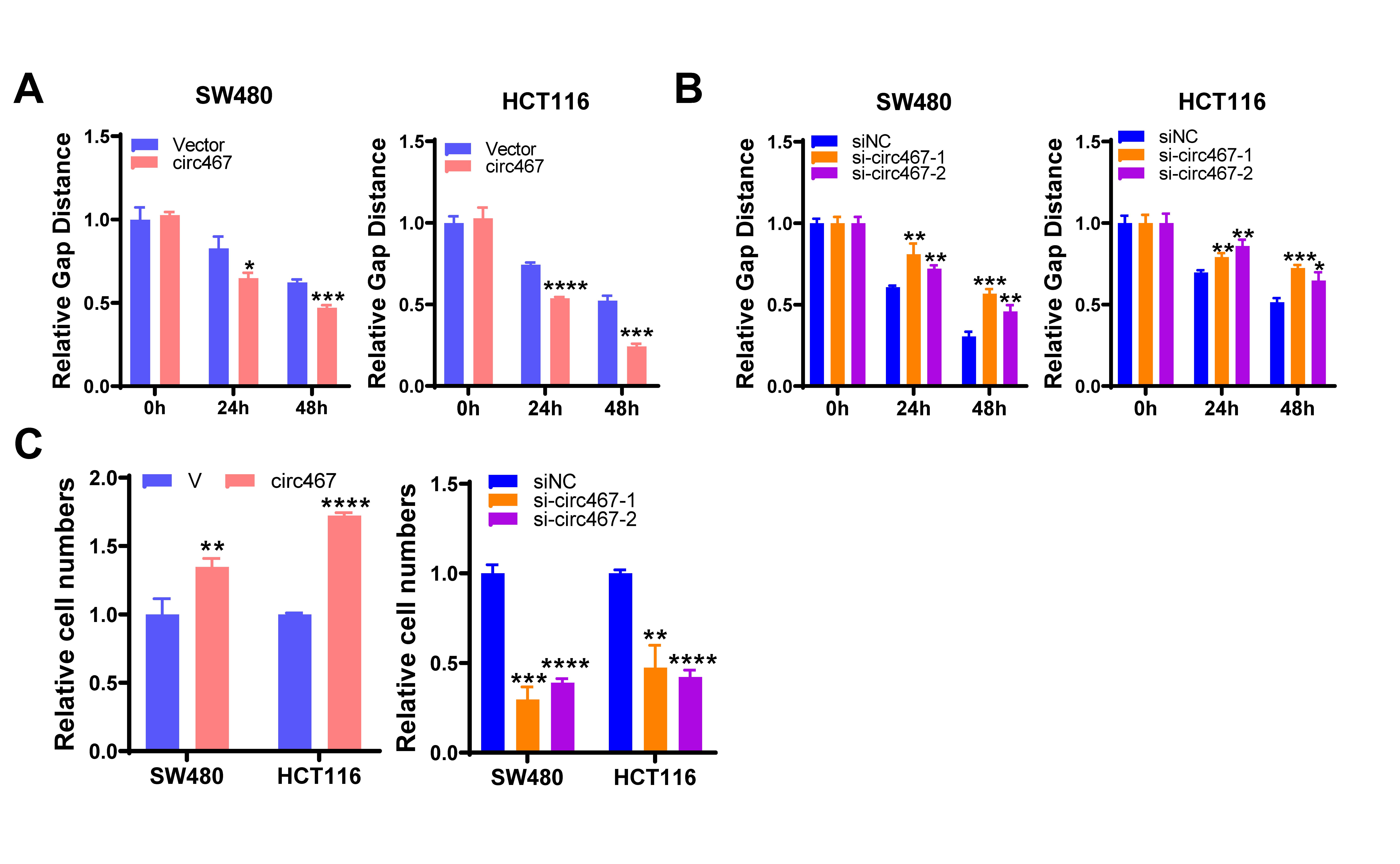

Supplement: Supplementary file 4 — Supplementary Material 4 [file 12943_2024_2052_MOESM4_ESM.jpg]

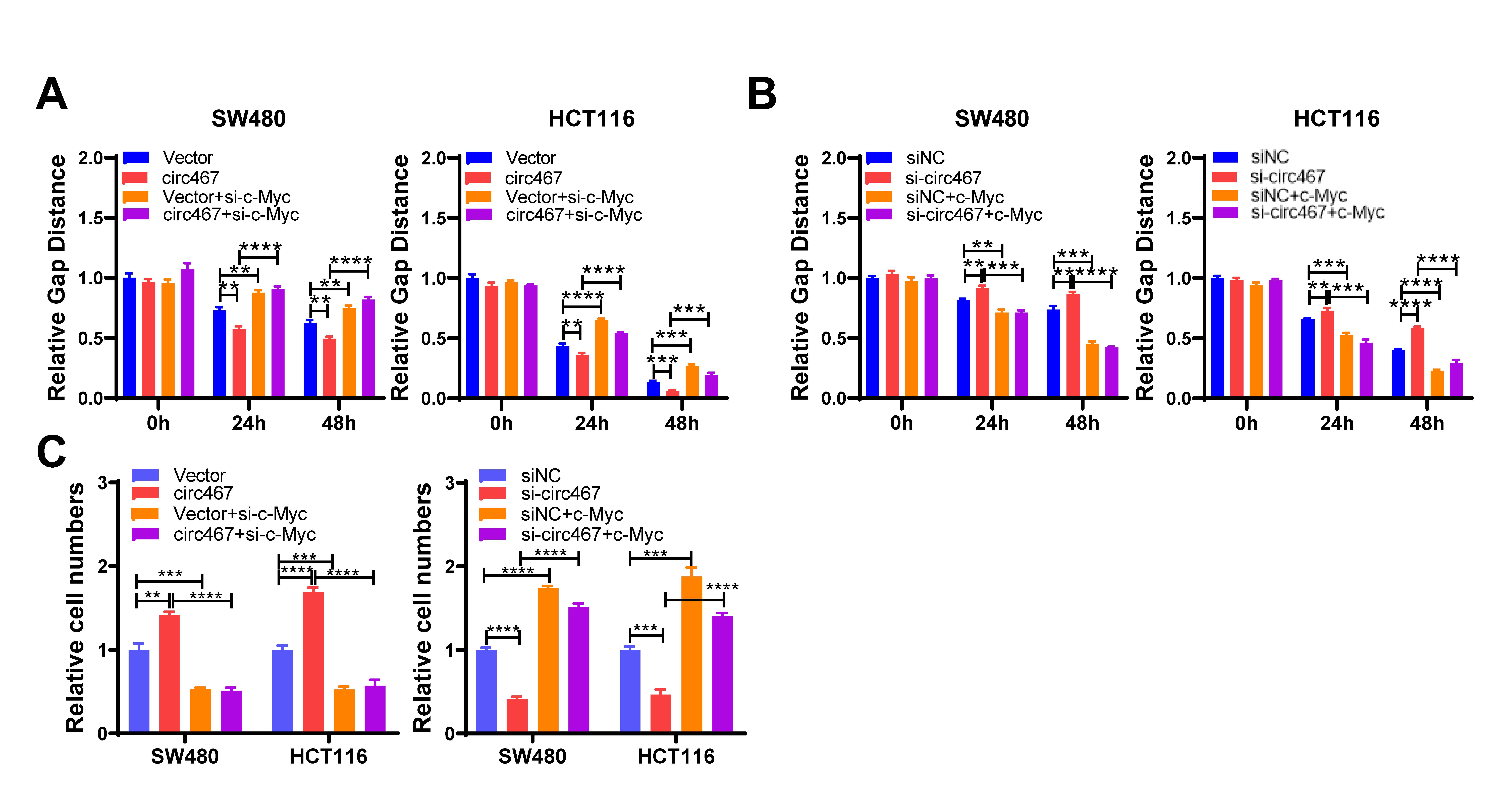

Supplement: Supplementary file 6 — Supplementary Material 6 [file 12943_2024_2052_MOESM6_ESM.jpg]
