# Supplementary material for: Circular RNA hsa_circ_0000467 promotes colorectal cancer progression by promoting eIF4A3-mediated c-Myc translation
Source: Mol Cancer. 2024 Jul 31;23:151. doi: 10.1186/s12943-024-02052-5 (PMC11290134; doi:10.1186/s12943-024-02052-5)
Supplement: Supplementary file 7 — Supplementary Material 7 [file 12943_2024_2052_MOESM7_ESM.docx]

**Supplementary Legends for**

Circular RNA hsa_circ_0000467 promotes colorectal cancer progression by promoting eIF4A3-mediated c-Myc translation

Xianjie Jiang, Mingjing Peng, Qiang Liu, Qiu Peng, Linda Oyang, Shizhen Li, Xuemeng Xu, Mengzhou Shen, Jiewen Wang, Haofan Li, Nayiyuan Wu, Shiming Tan, Jinguan Lin, Longzheng Xia, Yanyan Tang, Xia Luo, Qianjin Liao, Yujuan Zhou^#^

#**Correspondence to:** Yujuan Zhou, Hunan Key Laboratory of Cancer Metabolism, Hunan Cancer Hospital and The Affiliated Cancer Hospital of Xiangya School of Medicine, Central South University, Changsha, 410013, Hunan, China, 283 Tongzipo Road, Changsha 410013, Hunan, China. Tel: 86-731-88651681; Fax: 86-731-88651999; Email: [yujany_zhou@163.com](mailto:yujany_zhou@163.com)

**This file includes supplementary legends:**

Figures S1 to S6

Tables S1 to S3

**Supplementary Figures S1 to S6**

**Figure S1 Circ467 is highly expressed in CRC tissues and cell lines.**

A. Volcano plot showing differentially expressed circRNAs in the GSE138589 and GSE142837 datasets. B. Venn diagram showing 4 circRNAs that were differentially expressed in both the GSE138589 and GSE142837 datasets. C. Expression of the 4 circRNAs in CRC cell lines was determined using RT‒qPCR. The data shown are representative images or are expressed as the mean ± SD of each group from three separate experiments (*, *p* < 0.05; **, *p* < 0.01; ***, *p* < 0.001; ****, *p* < 0.0001 vs. control; Student’s t test).

**Figure S2. Associations between circ467 expression and the age (A), gender (B) histology grading (C), TNM stage (D) and metastasis (E) of CRC patients.** (*, *p* < 0.05; **, *p* < 0.01 vs. control; Student’s t test).

**Figure S3. Circ467 promotes the growth of CRC cells.**

A, B. Circ467 RNA expression in SW480 and HCT116 cells after circ467 overexpression or knockdown was measured using RT‒qPCR. C. Statistical analysis of the percentage of EdU-positive cells according to Figure 2C and 2D. D. Statistical analysis of the number of colonies according to Figure 2E and 2F. E. H&E staining was performed on the xenograft tissues, and circ467 and PCNA expression was assessed by *in situ* hybridization and immunohistochemistry, respectively. The data shown are representative images or are expressed as the mean ± SD of each group from three separate experiments or a single experiment (for the *in vivo* studies) (*, *p* < 0.05; **, *p* < 0.01; ***, *p* < 0.001; ****, *p* < 0.0001 vs. control; Student’s t test).

**Figure S4 Circ467 promotes the migration and invasion of SW480 and HCT116 cells.**

A. Quantification of migration according to the wound gap distance for SW480 and HCT116 cells after circ467 overexpression. B. Quantification of migration according to the wound gap distance for SW480 and HCT116 cells after circ467 knockdown. C. Quantification of the number of invading SW480 and HCT116 cells after circ467 overexpression or knockdown. The data shown are representative images or are expressed as the mean ± SD of each group from three separate experiments (*, p < 0.05; **, p < 0.01; ***, p < 0.001; ****, p < 0.0001 vs. control; Student’s t test).

**Figure S5 Circ467 promotes the proliferation of CRC cells by upregulating c-Myc.**

A. The expression of c-Myc in SW480 and HCT116 cells after cotransfection of circ467 plasmids and c-Myc siRNAs was measured using western blotting. B. The expression of c-Myc in SW480 and HCT116 cells after cotransfection of circ467 siRNAs and c-Myc plasmids was measured using western blotting. C. The percentage of proliferating SW480 and HCT116 cells after cotransfection of circ467 siRNAs and c-Myc plasmids was measured by EdU assay. D. The percentage of proliferating SW480 and HCT116 cells after cotransfection of circ467 plasmids and c-Myc siRNAs or circ467 siRNAs and c-Myc plasmids was measured by EdU assay. E. The colony formation abilities of SW480 and HCT116 cells after cotransfection of circ467 plasmids and c-Myc siRNAs or circ467 siRNAs and c-Myc plasmids were assessed by colony formation assay. The data shown are representative images or are expressed as the mean ± SD of each group from three separate experiments (*, p < 0.05; **, p < 0.01; ***, p < 0.001; ****, p < 0.0001 vs. control, Student’s t test).

**Figure S6 Statistics on the effect of circ467 expression and its regulation of downstream c-Myc on the invasion and metastasis of CRC cells**

A. Quantification of the gap width for SW480 and HCT116 cells after cotransfection of circ467 plasmids and c-Myc siRNAs. B. Quantification of the gap width in SW480 and HCT116 cells after cotransfection of circ467 siRNAs and c-Myc plasmids. C. Quantification of the number of invading SW480 and HCT116 cells after cotransfection of circ467 plasmids and c-Myc siRNAs or circ467 siRNAs and c-Myc plasmids. The data shown are representative images or are expressed as the mean ± SD of each group from three separate experiments (**, p < 0.01; ***, p < 0.001; ****, p < 0.0001 vs. control, Student’s t test).

**Supplementary Tables S1 to S3**

Table S1. Clinicopathological data of 36 paraffin-embedded adjacent nontumor epithelial tissues and 137 paraffin-embedded colorectal cancer tissues used for *in situ* hybridization (ISH)

Table S2. List of primer sequences for siRNA and RT‒qPCR and probe sequences for RNA pull-down experiments.

Table S3. List of antibodies used for western blotting, RNA immunoprecipitation and immunofluorescence (IF) experiments.
