# Supplementary material for: Circular RNA hsa_circ_0000467 promotes colorectal cancer progression by promoting eIF4A3-mediated c-Myc translation
Source: Mol Cancer. 2024 Jul 31;23:151. doi: 10.1186/s12943-024-02052-5 (PMC11290134; doi:10.1186/s12943-024-02052-5)
Supplement: Supplementary file 9 — Supplementary Material 9 [file 12943_2024_2052_MOESM9_ESM.docx]

**Table S2. List of primer sequences for siRNA and RT‒qPCR and probe sequences for RNA pull-down and ISH experiments.**

| **Symbol** | **Target sequence** |
| --- | --- |
| **siRNAs** |  |
| **Negative control (siNC)** | **UUCUUCGAACGUGUCACGUTT** |
| **si-circ467-1** | **AAAGUACACGAGCAAGAAG** |
| **si-circ467-2** | **UAAAAGTACACGAGCAAGA** |
| **si-c-Myc** | **GAGGAGACAUGGUGAACCA** |
| **si-eIF4A3-1** | **CGAGCAAUCAAGCAGAUCA** |
| **si-eIF4A3-2** | **GCTGGAUUACGGACAGCAU** |
| **RT-qPCR** |  |
| **Circ467-F** | **CGGTACATCGTATCCCAAGTTC** |
| **Circ467-R** | **CACAATTAGACAACTCTGGGTCAGA** |
| **hsa_circ_0000512-F** | **GGTCAGACTGGGCAGGAGATG** |
| **hsa_circ_0000512-R** | **GCCATTGAACTCACTTCGCTG** |
| **hsa_circ_0040809-F** | **CAACCCAGACGTGCAACAAAG** |
| **hsa_circ_0040809-R** | **CACTGCAATCTGAACCACATCG** |
| **hsa_circ_0084615-F** | **GTGATGGAGATTTTGATGTGGATG** |
| **hsa_circ_0084615-R** | **TGAAGAATGAAGTTCCTGAGAGTCC** |
| **c-Myc-F** | **CCTCACAGCCCACTGGTCCT** |
| **c-Myc-R** | **TGACCCTCTTGGCAGCAGGATAG** |
| **SKA3-F** | **TCACATTGCCCTGCCTCTG** |
| **SKA3-R** | **CTTAAAGAGTGAATGACTGGGCTAC** |
| **Probe** |  |
| **Circ467-probe** | **TTCTTGCTCGTGTACTTTTATTATTCCTCGCA** |
