# Supplementary material for: Circular RNA hsa_circ_0000467 promotes colorectal cancer progression by promoting eIF4A3-mediated c-Myc translation
Source: Mol Cancer. 2024 Jul 31;23:151. doi: 10.1186/s12943-024-02052-5 (PMC11290134; doi:10.1186/s12943-024-02052-5)
Supplement: Supplementary file 10 — Supplementary Material 10 [file 12943_2024_2052_MOESM10_ESM.docx]

**Table S3. List of antibodies used for western blotting, RNA immunoprecipitation and immunofluorescence (IF) experiments.**

| **Antibody** | **Catalog Number** | **Company** |
| --- | --- | --- |
| c-Myc | 5605S | CST |
| CyclinD2 | [A22261](https://abclonal.com.cn/catalog/A22261) | ABclonal |
| CDK4 | A23521 | ABclonal |
| ZEB1 | 3396T | CST |
| E-cadherin | 3195T | CST |
| eIF4A3 | 17504-1-AP | Proteintech |
| GAPDH | 60004-1-Ig | Proteintech |
